# Nanomaterials in Scaffolds for Periodontal Tissue Engineering: Frontiers and Prospects

**DOI:** 10.3390/bioengineering9090431

**Published:** 2022-09-01

**Authors:** Siyang Chen, Xin Huang

**Affiliations:** 1 Guanghua School of Stomatology, Hospital of Stomatology, Sun Yat-sen University, Guangzhou 510055, China; 2 Guangdong Provincial Key Laboratory of Stomatology, Sun Yat-Sen University, Guangzhou 510055, China

**Keywords:** tissue engineering, periodontal regeneration, nanomaterials, scaffolds

## Abstract

The regeneration of periodontium represents important challenges to controlling infection and achieving functional regeneration. It has been recognized that tissue engineering plays a vital role in the treatment of periodontal defects, profiting from scaffolds that create the right microenvironment and deliver signaling molecules. Attributable to the excellent physicochemical and antibacterial properties, nanomaterials show great potential in stimulating tissue regeneration in tissue engineering. This article reviewed the up-to-date development of nanomaterials in scaffolds for periodontal tissue engineering. The paper also represented the merits and defects of different materials, among which the biocompatibility, antibacterial properties, and regeneration ability were discussed in detail. To optimize the project of choosing materials and furthermore lay the foundation for constructing a series of periodontal tissue engineering scaffolds, various nanomaterials and their applications in periodontal regeneration were introduced.

## 1. Introduction

Known as a widespread infectious dental disease, periodontitis is characterized by the gradual destruction of periodontal tissues including cementum, periodontal ligament (PDL), and alveolar bone, ultimately resulting in tooth loss and negatively affecting masticatory function, aesthetics, and quality of life [1]. Although the etiology of periodontal defects is multifactorial, the subgingival dental biofilm is the most basic and important. It may destroy the periodontium irreversibly in a susceptible host, by eliciting a host inflammatory and immune response [2].

The ideal treatment for periodontitis is the functional regeneration of reduced tissues, which requires inserting organized PDL fibers into regenerating cementum and adjacent alveolar bone and ultimately reconstructing the composite periodontium [3]. However, traditional treatments like guided tissue regeneration (GTR), are efficient at improving periodontal treatment outcomes (like increasing attachment gain, reducing pocket depth, preventing further gingival recession, etc.) but do not predictably achieve functional regeneration [1]. It remains a significant hurdle to regenerate periodontium because of the lack of spatiotemporal healing coordination, and long-term effective anti-bacterial and regenerative stem cells [4]. It is promising to apply tissue engineering in periodontal regeneration to overcome such obstacles [5]. As a core component of these approaches, scaffolds can facilitate regeneration by managing the complex spatiotemporal events during the healing of periodontitis [6]. However, the existing bulk biomaterial scaffolds often fail to achieve the desired effect due to the lack of integration with the host tissue, inadequate antibacterial properties, and insufficient bioactivity [7]. To improve the listed problems, experts have been working on new technologies and materials. Based on the advancement of nanotechnology, nanomaterials have become popular as multi-functionalized scaffolds for periodontal tissue engineering, which helps to mimic the extracellular matrix (ECM), resist bacterium, and promote stem cell differentiation [8,9,10].

To give a general overview of nanomaterials in scaffolds for periodontal tissue engineering, this review summarized the properties of current nanomaterials and nanoscaffolds. It also listed a series of nanomaterials with excellent properties and their application in successful tissue formation for periodontal regeneration, which may have critical references for the progress of periodontal tissue engineering and periodontitis treatment.

## 2. Tissue Engineering and Scaffolds

Generated the knowledge from engineering and life sciences, tissue engineering aims at obtaining components similar to those present in life, and it helps to maintain, improve or recover the function of various organs and tissues when it is used in the organism [11]. Tissue engineering consists of three main factors: (1) cells, as the basic structural unit of all tissues; (2) scaffolds, as frameworks supporting the activities of cells to form a complete organism; and (3) biological factors like growth factors and specific protein facilitating cellular activity [12]. A successful tissue engineering process depends on manipulating gene expression and cell interactions, selecting a scaffold material that precisely mimics native tissue structure, ultimately restoring relative function. Because the scaffolds are related to the microenvironment for cell adhesion, growth, proliferation, differentiation, and formation of the new tissues, thus the selection of scaffold materials and the design of scaffold construction are keys to realizing optimal periodontal regeneration and improving the efficiency of periodontitis treatment [13].

Ideal tissue engineering scaffolds should have some features: (1) biological properties: be biocompatible to avoid immune responses and biodegradable to help the formation of new organizations [14,15], partially simulate the components, and structure of the ECM [16]. (2) physical properties: have appropriate porosity and pore size to aid tissue vascularization and integration, have sufficient mechanical properties to supply a proper environment for cells, distribute cells or inductive materials to the wounding location, and offer cues to the restoration of composition and function of newly created tissue [17,18]. (3) chemical properties: have proper surface chemistry to allow cell adhesion, proliferation, and differentiation [10]. The general scaffolds, however, have a few inherent drawbacks, such as a lack of antibacterial properties, mechanical instability, and poor ability to induce tissue regeneration [13]. To design scaffolds with ideal characteristics, like biocompatibility, antibacterial properties, and capacity to enhance cell activities for the organized regeneration of the entire alveolar bone-PDL-cementum complex, nanomaterials have attached increasing importance to their advanced characteristics.

## 3. Properties of Nanomaterials

As described above, to provide a proper microenvironment for both cell adhesion, proliferation, differentiation, and forming the new tissues, ideal tissue engineering scaffolds should possess a range of qualities. Nanoscale materials that range in size from 1 to 100 nm are called nanomaterials [7]. Nanomaterials show unique physicochemical and biological properties compared to their larger-scale form. For example, nanomaterials ensure smaller sizes, larger surface-to-volume ratios, and more reactive properties with cells [19]. Moreover, due to the development of nanotechnology, nanomaterials have allowed the production of scaffolds possessing properties proximity to ideal ones which opened a new era for tissue engineering [20,21,22]. Several advantages of nanomaterials, which are beneficial to the process of tissue engineering, are detailed below, including favorable biocompatibility, antibacterial properties, and regeneration ability [23].

### 3.1. Superior Biocompatibility

Periodontium is a complexion composed of soft and hard tissue meaning that it is an intricate system. It consists of periodontal ligament (PDL), cementum, and alveolar bone. Since tissue engineering has been applied in periodontitis treatment and periodontal regeneration over the last few years, scaffolds based on different materials have been designed. These scaffolds are active in periodontal regeneration, while it might be difficult for them to mimic the natural periodontal microenvironment which limits their roles in the reconstruction of tooth-supporting tissue.

Extracellular matrix (ECM), produced by the cells, is a component of biomolecules that comprises a specific tissue. As a well-hydrated nanocomposite, typical ECM contains various proteins (signaling proteins, adhesion proteins, proteoglycans, etc.) and fibers (collagen fibers, reticular fibers, elastic fibers, etc.), most of which contribute to its rigidity [23]. Besides, a specifically mimicked native ECM scaffold has been shown to promote cytoskeleton arrangement, cell signaling, migration, growth, adhesion, and differentiation [23,24,25].

Since there are some nanoscale components in the ECM, the structures of nanoscale scaffolds are closer to the natural ECM than that of the larger-scale scaffolds. Nanomaterials can be created to be extremely proximity to specific ECM to promote cell migration, adhesion, and growth in damaged tissues [26]. It has been found that tissue engineering scaffolds that closely simulate porosity and surface area of natural ECM with nano-size provide a microenvironment promoting cell responses, including adhesion, proliferation, and differentiation [27]. Both in vitro and in vivo studies have shown that a nanofibrous topography promoted precursor cells to differentiate into osteoblasts and odontoblasts [24]. Besides, the fibrous topography can also facilitate the expression of particular signaling molecules triggering cellular differentiation [24].

### 3.2. Superior Antibacterial Properties

Periodontitis is an infectious disease caused by bacteria that leads to irreversible destruction of the periodontium. Topical antibiotics therapy is the base of periodontal treatment strategy and tissue regeneration, but the limited properties of current antibiotics inhibit the curative effects severely [28]. Plaque biofilm is a complex community of bacteria, which is a major pathogenic factor of periodontitis, caries, and other oral diseases. It protects pathogenic microorganisms from both the host defense mechanisms and external drug agents resulting in infection [28]. Numerous studies have been conducted to design antimicrobial agents to solve such problems, but few of them get the desired outcomes because fast release and the rapid degradation of antibacterial medicines bring on safety concerns and low efficiency. Therefore, it is important to develop long-term antibacterial materials to manage the inflammatory environment in periodontal treatment and tissue regeneration.

Thanks to the unique physicochemical properties like large surface-area-to-mass ratio, ultra-small sizes, and positive chemical reactivity, nanomaterials are promising in antibacterial therapies. Nanoparticles (NPs) possess larger surfaces and higher charge density, which allows them to interact with negatively-charged surfaces of bacterium more effectively than their larger-scale particles, ultimately enhancing antimicrobial properties [29]. Metallic and organic NPs have been introduced to a few areas of dentistry because they have a good antibacterial effect against the broad-spectrum bacterium. Moreover, the antibacterial effect of NPs correlated with their size so smaller NPs gained more popularity by releasing more corresponding ions [30]. Besides, various experimental and clinical research concentrated on the antibacterial effect of NPs confirmed that NPs showed great antibacterial properties in resistant bacteria. Therefore, the application of NPs in periodontal tissue engineering might obtain potential advantages.

### 3.3. Superior Regeneration Ability

Without a doubt, the healing of periodontal tissue is impeded by bacterial infection, while the other main element influences the overall outcome of periodontal regeneration is restricted bone regeneration. There are not sufficient residual periodontal ligament cells (PDLCs) to achieve complete periodontal regeneration, due to the destruction of periodontitis to relative cells. Thus, to overcome the shortcomings of conventional ones, it is urgent to develop scaffolds incorporated with antibacterial and osteogenic functions [31].

As pluripotent differentiated cells, PDLCs are potential native cell sources for the complete regeneration of periodontium. However, the differentiation capacity of PDLCs is affected by functional regulation in periodontal regeneration [32]. To facilitate the cell activities of PDLCs, many kinds of promising regenerative agents have been applied in periodontal tissue engineering, which includes those based on polymeric and inorganic NPs. Due to the larger surface-to-volume ratio, NPs can better promote the interaction with biological molecules and cells compared to their larger-scale particles. For example, it has been demonstrated that gold nanoparticles (AuNPs) are effective in stem-cell osteogenic differentiation [33]. Hydroxyapatite (HA) with the chemical formula of Ca_10_(OH)_2_(PO_4_)_6_, is a biocompatible and highly hydrophilic inorganic, which possesses excellent osteoconductivity and good hydrophilicity [34]. Nanosilicate (nSi, Na^+^_0.7_ [(Mg_5.5_Li_0.3_)Si_8_O_20_(OH)_4_]^−^_0.7_), incorporated into poly (lactic-co-glycolic acid) (PLGA) fibers, can dissociate into nontoxic ionic products (Na^+^, Mg^2+^, Si(OH)_4_^+^, Li^+^) in aqueous solutions, which can regulate cellular responses associated with periodontal regeneration. In addition to silicate being an indispensable component in the process of the new bone formation, other ions (Mg^2+^, Si(OH)_4_, Li^+^) have also been demonstrated to induce osteogenic effects via several signaling pathways [35].

## 4. Nanomaterials Applied in Periodontal Tissue Engineering

### 4.1. Nanofibers

A great deal of interest has been shown in developing nanofibers for periodontal tissue engineering due to their ability to mimic native networks, which are the main elements of typical ECM. Nanofibers, possess the capacity for reconstructing the architecture of native ECM, and can provide proper scaffolds for cell adhesion and other essential activities [36]. In addition, nanofibers can also offer an ideal microenvironment for the spread of bioactive factors due to the high specific surface area. Therefore, nanofibrous scaffolds, equipped with unique characteristics including controllable porosity with interconnected pores and a high surface area-to-volume ratio, play significant roles in periodontium regeneration by enhancing protein absorption, activating specific gene expression and signaling within cells, and promoting cellular processes [10].

#### 4.1.1. Nanomaterials Incorporated into Nanofibers

To create ideal scaffolds suitable for periodontal regeneration, a series of polymers and active bioceramics, have been incorporated into nanofibers (as shown in Table 1). For example, due to the favorable biological properties of natural polymers (like chitosan, bacterial cellulose, gelatin) and the favorable mechanical properties of synthetic polymers (like poly-caprolacton, poly (lactic acid-co-glycolic acid), polylactic acid), nanofiber scaffolds are often incorporated with these two nanomaterials to maximize their benefits. Inorganic ceramics like nano-hydroxyapatite and bioactive glasses are proposed to promote the process of bone regeneration, which is also possible to improve physical properties at the same time.

#### 4.1.2. Advanced Techniques Fabricated Nanofibers

It has been possible to fabricate nanofibers using varieties of technologies, including phase separation, self-assembly, wet spinning, dry spinning, and electrospinning [41]. The fabrication of nanofibers using electrospinning has become increasingly popular in some applications such as tissue engineering and drug delivery because it allows for simple and continuous fabrication. Moreover, it can produce nanoscale materials with controllable and ideal fiber diameters, porosities, morphologies, and surface properties [13].

A typical electrospinning setup requires four elements: a syringe pump, a metallic needle spinneret, a power supply with high voltage, and a grounded conducting collector [13]. Recently, researchers have successfully obtained aligned fibers instead of traditional random thin ones by altering the collector, providing extra electric or magnetic fields. As well, several studies found that many kinds of stem cells, like human gingival fibroblasts, human mesenchymal stem cells (hMSCs), and dental follicle stem cells, responded more synergistically to gingival regeneration signals in chemical and topological pathways in the aligned scaffold group compared to the random scaffold group [53,54,55]. In this regard, the ordered electrospun nanofibers may be better suited to periodontal regeneration, especially the regeneration for PDL.

Thanks to computer-aided design (CAD) and three-dimension(3D) printing techniques, integrated periodontal regeneration was achieved with multiphase and region-specific nanofiber scaffolds that delivered bioactive cues spatiotemporally [56]. For example, a multilayered PCL and Sr-nHA porous structure, on the basis of an innovative architectural design and the formulations of established biomaterial, was developed to regenerate periodontal hard tissue. Finally, the biocompatibility and osteogenic potential of human osteosarcoma cells was evaluated in vitro biological performance. The study in vitro suggested that the ceramic phase of the polymeric matrix seems to play a significant role in bone mineralization [57].

Furthermore, it has been demonstrated that 3D printing can be utilized for fabricating multilayered scaffolds with heterogeneous properties semblable to native tissues. According to reported findings, a variety of 3D printing scaffolds designed for regenerative purposes can benefit from nanoscale features. Therefore, future studies in periodontal tissue engineering may focus on combining nanotechnology and 3D printing to manufacture region-specific nanomaterial-based scaffolds that deliver bioactive cues spatiotemporally for integrated periodontium regeneration.

### 4.2. Antibacterial Nanomaterials

Antibacterial nanomaterials incorporated into electrospun nanofibers have the potential to provide a controllable antibacterial function which is a benefit for periodontal regeneration activity [13]. Various metallic NPs with superior antibacterial properties are extensively used in periodontal tissue engineering, which helps to enhance the properties of nanofibers. Metallic NPs, such as silver (Ag), zinc oxides (ZnO), titanium dioxide (TiO_2_), and magnesium oxides (MgO) are able to interfere with the infection process of bacteria in varieties of methods. For example, it can destroy cell membranes and mitochondrial and denature proteins by disrupting pathogen membranes and generating reactive oxygen species (ROS) [58]. Additionally, the mechanisms of antimicrobial activity include disturbance of metabolic processes, disruption of macromolecular oxidation, replacement of magnesium ions needed for enzymatic activity in oral biofilms, electron delivery, and inhibition of DNA replication [28]. Besides, to overcome the bacterial resistance associated with biofilm formation by pathogens, metallic NPs have widely been studied as agents for penetrating the biofilm and reducing bacteria.

Metallic NPs have been demonstrated to be beneficial in treating periodontitis as antimicrobial agents in a variety of applications, thereby preventing infection from occurring (as shown in Table 2). AgNPs, with good biocompatibility, have possessed a better antimicrobial efficiency compared to other antimicrobial molecules. As well as introducing the antibacterial activity, ZnO NPs have the potential to improve osteoconductivity. MgO NPs, as light metal-based NPs, show high antibacterial capacity which can be fully resorbed in the body. TiO_2_ NPs have been incorporated into various polymer patches which showed markable antibacterial ability against gram-positive and gram-negative bacteria.

Apart from the metallic NPs mentioned above, as a derivative of graphene, graphene oxide (GO) is a promising antimicrobial nanomaterial [32]. For example, He and collaborators reported that GO nanosheets decreased the numbers of gram-negative anaerobic bacteria, related to periodontitis when they were present in the presence of GO nanosheets [59]. However, researches on graphene-based nanomaterials with antibacterial properties in periodontal tissue engineering are still insufficient. Future research on graphene-based nanomaterials can take advantage of their various antibacterial mechanisms.

### 4.3. Nanomaterials for Regeneration

To ensure biocompatibility, a material used for scaffolds in dentistry should be able to maintain the native environment of the healthy oral cavity. Additionally, it is required to be beneficial for stem cells to grow, proliferate and differentiate into specific-lineage tissue. It has been proven that graphene-based nanomaterials applied in dentistry have the potential to stimulate cellular biomineralization and osteogenic differentiation due to their osteoconductivity. Besides, several metallic nanomaterials induced the osteogenic effect depending on the activation of various signaling pathways [68] (as shown in Table 3).

Bone regeneration plays an essential part in periodontal regeneration. Researchers have found that the incorporation of graphene-based nanomaterials into scaffolds can facilitate osteoconductivity properties by the stimulation of osteogenic differentiation and biomineralization in cells [32]. It is worth noting that metallic nanomaterials also have a significant impact on cell differentiation when being introduced into scaffolds for tissue engineering.

### 4.4. Nanomaterials for Drug Delivery Systems and Other Potential Applications

Currently, nanoscale drug delivery systems have attracted great attention in the treatment of oral diseases, which can improve therapeutic outcomes and reduce unnecessary side effects. Various studies have shown the benefits of applying nanoscale delivery systems to periodontitis. In addition, nanomaterials have potential clinical applications in targeted therapeutics for periodontitis, monitoring in the process of periodontitis, and so on.

#### 4.4.1. Nanomaterials for Drug Delivery Systems

The infection and lack of growth factors often lead to the failure of periodontal regeneration therapy. However, the scaffolds commonly used in periodontal tissue engineering have excellent biocompatibility but have limitations in promoting regeneration and antibacterial properties. Therefore, it is important to develop scaffolds for controllably and sustainedly delivering active ingredients or drugs in periodontal defects. Recently, mesoporous nanoparticles (MNs), with a pore diameter of 2–50 nm, have attracted increasing attention as periodontal delivery systems, which possess controllable physicochemical properties and high surface-to-volume ratios for loading kinds of active ingredients [76]. Mesoporous silica nanoparticles (MSNs) have been the most widely used carriers among numerous MNs because of some specific advantages [28].

Due to their favorable physical properties (such as high specific surface area, large pore volume, multiple channels, and designable shapes), chemical properties (easy surface modification), and good biocompatibility, MSNs succeed in prolonging drug release. Tan etc. designed a MSNs drug delivery system for carrying resveratrol to enhance its stability and prolong its duration which led to a favorable therapeutic efficacy in diabetic periodontitis by regulating the polarization of the macrophage [77]. Another study constructed nanocomposite membranes that could deliver moxifloxacin by incorporating MSNs particles into PLGA fibers. The drug release is significantly prolonged because the drug needs to be released from the MSNs to the PLGA fibers first and then diffused from the fibers into the medium [78].

#### 4.4.2. Nanomaterials for Other Potential Applications

Studies have found that antimicrobial photodynamic therapy (aPDT) possesses potent antibacterial properties against target oral bacteria. Combining NPs with traditional photosensitizers can improve the current problems, such as the low solubility in aqueous media and unstable in vivo performance, enabling more efficient and widespread clinical applications of aPDT. Therefore, NP-based aPDT has become a hot topic in the antimicrobial treatment of oral diseases [79]. For example, the photosensitizer Ce6 loaded upconversion NPs (NaYF4:Yb,Er) showed remarkable antibacterial outcomes on Porphyromonas gingivalis, Prevotella intermedia, and Fusobacterium nucleatum under 980 nm irradiation, exhibiting a potential application in the management of periodontitis [80].

Currently, the clinical diagnosis of periodontal disease mainly includes clinical examination and imaging evaluation. Gingival crevicular fluid (GCF) contains many components, such as electrolytes, proteins, some cells, and enzymes, whose changes can reflect the process of periodontal destruction, so the detection of gingival crevicular fluid shows a high diagnostic potential [81]. The concentration of electrolytes in GCF can reflect the health or disease state of periodontal tissue, so these ions can be used as potential diagnostic markers for periodontal disease states. A series of small-scale sodium-selective membranes with magnetic nano-inclusions were developed, which can be used as sodium-selective sensors to measure ions in GCF. These new devices could enable early diagnosis and prevention of periodontal disease by detecting changes in inorganic ion levels in GCF [82].

## 5. Synthesis Methods of Nanomaterials

There are generally two types of synthetic pathways for nanomaterials, top-down methods which are to decompose bulk materials into nanomaterials, and bottom-up methods which are to build nanomaterials from many atoms [83]. Various methods of fabricated nanomaterials can be divided into traditional methods and green synthesis methods. Traditional synthetic processes include thermal decomposition, solvent evaporation, polyol process, liquid–liquid interface, emulsion diffusion, electrochemical methods, and so on [84]. Though these traditional synthetic methods enable efficient fabrication of nanomaterials, toxic waste and by-products may be generated during the synthesis. These have forced researchers to turn their attention to green synthesis approaches.

Green synthesis methods are the application of natural biological systems to nanomaterial production, which can avoid the above-mentioned shortcomings of the traditional synthesis methods and realize an environmentally friendly production process. Several microorganisms (including bacteria, yeast, and fungi), algae, and some specific plants have been utilized as substrates for green synthesis [85]. Since this synthetic method is not yet used in periodontal tissue engineering, it has great potential application value in periodontal regeneration therapy.

## 6. Potential Toxicity of Nanomaterials

Nanomaterials are similar in size to the biological molecules, DNA, and proteins of cells and most viruses, which means they are capable of traveling throughout the body, depositing in certain organs, penetrating cells, hanging about in the mitochondria, ultimately provoking injurious responses [86]. It has been confirmed that several kinds of NPs, such as Ag, ZnO, TiO_2_, MgO, and aluminum oxide, are able to accumulate in the vital systems like circulatory systems, digestive systems, respiratory systems, and urinary systems in animal experiments and may have similar adverse effects on human bodies [87]. A number of mechanisms are involved in the toxic effects of NPs in their target organs, which include generating reactive oxygen species (ROS), causing DNA damage, altering protein structures, and functions, and disrupting membrane integrity [88,89]. For example, it has been shown that incorporating AgNPs into periodontal scaffolds enhanced the mechanical properties and antibacterial features of the constants. However, AgNPs perform potential toxicity due to the free silver ions which may result in the damage of biological molecules, cells, and tissues. According to several types of laboratory research, AgNPs can impair mitochondrial function and induce oxidative stress in humans [60]. Thus, the potential toxicity of AgNPs still needs to be investigated because their clinical significance is unknown.

Although nanomaterials would bring unquestionable benefits to the treatment of periodontal disease, the introduction of these man-made nanomaterials may also pose potential safety risks. Therefore, the risk must be minimized in the process of designing new materials. This requires a careful selection of nanomaterials in terms of biocompatibility and in vivo stability. It is promising that with the progress of research, more safe and efficient nanomaterials will be developed and applied in clinical periodontitis treatment.

## 7. Conclusions & Future Perspective

In conclusion, with favorable biocompatibility, antibacterial properties, and regeneration ability, nanomaterials are promising in periodontal tissue engineering. Acting as advanced materials in scaffolds for periodontal tissue engineering, nanomaterials possess some ideal properties which allow them to achieve a better periodontal repair effect compared with conventional materials. In the next phase, quantities of nanomaterial-based multiphase scaffolds, closer to ideal tissue constructs, will be developed to facilitate the re-establishment of the architecture and function of the native periodontium.

Despite the significant advances in scaffold nanomaterials in the field of periodontal tissue engineering, it is still necessary to overcome numerous challenges. First, the potential toxicity of nanomaterials is an inevitable scientific problem, so long-term evaluation and observation are needed in vivo, not just in vitro or at the cellular level. Second, nanomaterial-based scaffolds are not optimum solutions for every current problem, which may be solved by integrating a hierarchical design covering a wide range of lengths. Third, 3D printing could help to develop nanomaterials-based scaffolds more appropriate to the actual periodontal condition, thus it might assume a more important role in periodontal tissue engineering in the future.

## Figures and Tables

**Table 1:** List of selected polymers and bioceramics in nanofibers for periodontal regeneration.

	Merits	Limitations	Potential Applications	Ref.
Natural polymers				
Chitosan (CS)	biodegradable, biocompatible, nontoxic, biologically renewable, bacteriostatic	little solubility in organic solvents and neutral aqueous solutions	Chitosan-based scaffold promoted human gingival fibroblasts and osteoblasts metabolism and mineralization. Chitosan NPs promoted the osteogenic differentiation of ^1^ BMSCs.	[37,38]
Bacterial cellulose (BC)	biocompatibility, low cost, ease of processing, ideal mechanical properties like high tensile strength	handicap in quality control related to contaminations	The non-resorbable BC membrane helped the closure of the class II furcation lesions in humans. The commercial BC membrane led to sufficient ^2^ GTR outcomes in human periodontal defects.	[39,40]
Structure protein	great biological properties like biocompatibility, resorbability, enhancing cell adhesion	immunoreactivity associated with its bovine source and allogeneic species	^3^ TSF enhanced the mesenchymal stem cell differentiation toward osteoblasts. Core-shell nanofibers utilizing zein prolonged metronidazole release.	[41,42]
Gelatin (GEL)	ideal biocompatibility, low immunogenicity	dissolubility in organic solution	GEL possessed bio-signal groups to enhance the proliferation of ^4^ hPDLSCs. The incorporation of GEL into nanofibrous membranes increased the osteogenic capability of preosteoblasts.	[43,44]
Alginates	biocompatible, hydrophilic, non-immunogenic, cost-effective	poor cell adhesion, low mechanical strength, low degradability	Alginates particles in hybrid scaffolds provided an early release of IGF-1 and BMP-6. ^5^ RGD-modified alginate scaffold enhanced MSC viability and osteogenic differentiation.	[45,46]
Synthetic Polymers				
Polylactic acid(PLA)	high mechanical strength	hydrophobicity, cause inflammation	The nHA/Collagen/PLA scaffolds promoted ^6^ hAMSCs seeding, proliferation, and osteogenic differentiation. Pure PLA nanofibers scaffolds facilitated BMSCs proliferation.	[37,47]
Poly (lactic acid-co-glycolic acid) (PLGA)	biocompatible, biodegradable	weak hydrophilicity, cell adhesion, acidic degradation products	The PLGA particles in hybrid scaffolds provided a lasting release of IGF-1 and BMP-6. The ^7^ DMOG/nSi-PLGA fibrous compounds enhanced and orchestrated osteogenesis-angiogenesis.	[35,45]
Poly-caprolactone (PCL)	enhanced mechanical properties, proper degradation kinetics with morphological characteristics	poor hydrophilicity, cause inflammation	The hybrid PCL scaffolds promoted PDLC differentiation and periostin expression. The electrospun PCL membranes presented a controlled release profile of the active compounds induced fibroblast formation.	[48,49]
Bioceramics				
Hydroxyapatite (HA)	Bioactive, biocompatible, excellent mechanical properties	poor degradation rates, drug release properties	HA-based coil scaffolds promoted angiogenesis and osteogenesis in rat and rabbit critical-sized bone defection. The magnesium-doped and the bromelain-functionalized HA-based scaffold regenerated periodontal tissue in vivo in a Wistar rat model.	[34,50]
Bioactive glass (BG)	facilitate growth factor production, gene expression, the proliferation of osteoblasts, and reconstruction of bone tissue	commercial BGs only show bone formation, without cementum or PDL	The nBG in the PCL composite scaffold enhanced the adhesion, and proliferation of hPDLCs. The fish collagen/bioactive glass/chitosan nano-composite scaffold promoted the formation of new bone and light inflammation occurred in the beagle dog’s periodontal defect model.	[51,52]

^1^ BMSCs, bone marrow stem cells; ^2^ GTR, guided tissue engineering; ^3^ TSF, tussah silk fibroin; ^4^ hPDLSCs, human periodontal ligament stem cells; ^5^ RGD, arginine-glycine-aspartic acid tripeptide; ^6^ hAMSCs, human amnion mesenchymal stem cells; ^7^ DMOG, dimethyloxalylglycine.

**Table 2:** List of metallic NPs as antimicrobial agents in periodontal regeneration.

	Materials	Potential Applications	References
Ag	chitosan-AgNPs	inhibited the growth of Porphyromonas gingivalis and Fusobacterium nucleatum related to dose	[60]
	AgNPs synthesized with an appropriated capping agent	promoted gram-negative bacterial inhibition	[58]
	AgNPs	possessed an anti-inflammatory effect by modulating inflammatory cytokines and regenerating growth factors	[61]
	the ^1^ PP-pDA-COL-Ag scaffold	promoted alveolar bone regeneration and accelerated periodontitis treatment in a mouse periodontitis model	[62]
ZnO	chitin hydrogel-ZnO	exhibited osteogenesis promotion both in vitro and rat periodontal defect model in vivo	[63]
	PCL/GEL-ZnO	decreased the number of planktonic and the formation of the Staphylococcus aureus biofilm	[64]
MgO	HA/^2^ PLLA-nMgO	enhanced osteoblast adhesion and proliferation	[65]
	^3^ PLA/gelatin-nMgO	guided periodontal tissue regeneration in rat periodontal defect models	[66]
TiO_2_	^4^ P(VDF-TrFE)- TiO_2_ nanowires	increased fibroblasts and osteoblasts adhesion and proliferation	[67]

^1^ PP-pDA-COL-Ag, PLGA/PCL-polydopamine-collagen-Ag; ^2^ PLLA, poly (l-lactic acid); ^3^ PLA, poly-caprolactone; ^4^ P(VDF-TrFE), poly(vinylidene fluoride-trifluoroethylene).

**Table 3:** List of nanomaterials for regeneration in periodontal tissue engineering.

	Materials	Potential Applications	References
graphene-based nanomaterials	^1^ PHB/1%CNTs scaffolds	enhanced attachment and proliferation of the ^2^ PDLSCs	[69]
	^3^ GO scaffolds	promoted cellular ingrowth behavior and the formation of dog bone defect	[70]
	^4^ PGO/HA-AG scaffolds	induced alveolar bone regeneration in bone defects of diabetic rat periodontitis models	[71]
	^5^ PCL -GO composites	promoted the proliferation of ^6^ hPDLSCs moderately, favored the differentiation of osteogenic	[72]
Metallic NPs	^7^ poly(LLA-co-CL)/nDPs scaffolds	enhanced seeding efficiency of ^8^ BMSCs	[33]
	Human β-defensin 3 ^9^ AuNPs	promoted the osteogenic differentiation of ^10^ hPDLCs in inflammatory microenvironments	[73]
	L/D-cysteine anchored AuNPs	facilitated osteogenic differentiation into hPDLCs and regenerating alveolar bones and periodontal ligaments in rat periodontal-defect models	[74]
	PCL or PCL/gelatin-nCaO matrices	increased the viability and osteogenic differentiation of osteoprecursor cell	[75]

^1^ PHB/CNTs, poly (3-hydroxybutyrate)/1% carbon nanotubes; ^2^ PDLSCs, periodontal ligament stem cells; ^3^ GO, graphene oxide; ^4^ PGO/PHA-AG, polydopamine-mediated graphene oxide/hydroxyapatite nanoparticle- alginate/gelatin; ^5^ PCL, poly-caprolactone; ^6^ hPDLSCs, human periodontal ligament stem cells; ^7^ LLA-co-CL/nDPs, l-lactide-co-ε-caprolactone/nanodiamond particles; ^8^ BMSCs, mesenchymal stem cells; ^9^ AuNPs, gold nanoparticles; ^10^ hPDLCs, human periodontal ligament cells.

## Data Availability

Not applicable.
